# Supplementary material for: Identification and Validation of a New Peptide Targeting Pancreatic Beta Cells
Source: Molecules. 2022 Mar 31;27(7):2286. doi: 10.3390/molecules27072286 (PMC9000318; doi:10.3390/molecules27072286)
Supplement: Supplementary file 1 [file molecules-27-02286-s001.zip › molecules-1615704-supplementary.pdf]

molecules

MDPI

Supplementary Materials

Identification and Validation of a New Peptide Targeting Pancreatic Beta Cells

Qianwen Wang [1], Lei Zheng [2], Kangze Wu [3] and Bo Zhang [3,*]

[1] Department of Surgery, Fourth Affiliated Hospital, School of Medicine, Zhejiang University, Yiwu 322000, China; 22118074@zju.edu.cn
[2] Department of Emergency Surgery, the Affiliated Hospital of Qingdao University, Qingdao 266000, China; 11718338@zju.edu.cn
[3] Department of Surgery, Second Affiliated Hospital, School of Medicine, Zhejiang University, Hangzhou 310009, China; wukangze@zju.edu.cn
* Correspondence: jjs10@zju.edu.cn

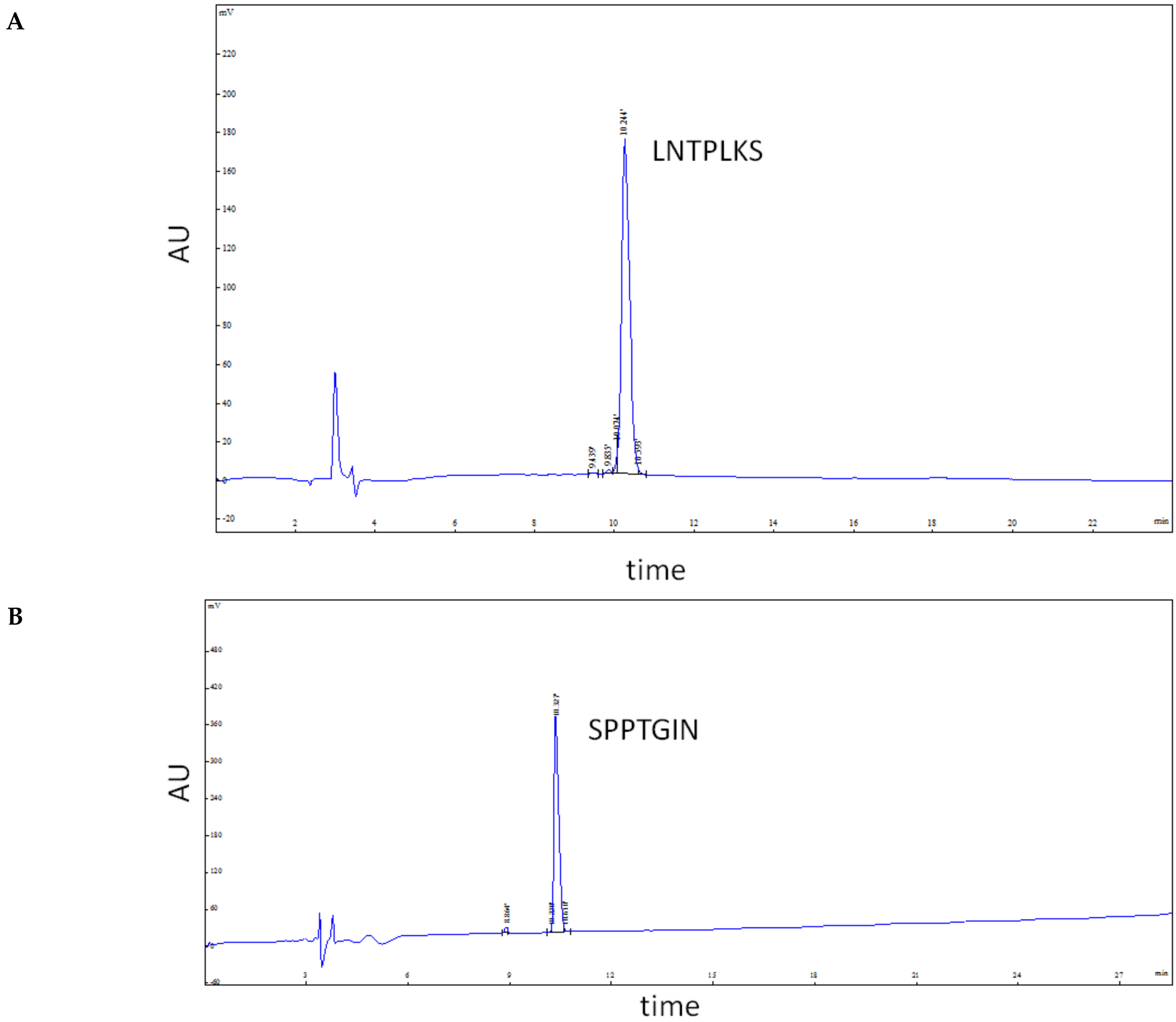

Figure S1. HPLC profiles of LNTPLKS (**A**) and SPPTGIN (**B**) peptide, showing that the purity of both peptide greater than 95%.
